# Brain Imaging of Human Sexual Response: Recent Developments and Future Directions

**DOI:** 10.1007/s11930-017-0123-4

**Published:** 2017-10-23

**Authors:** Gerben B. Ruesink, Janniko R. Georgiadis

**Affiliations:** Department of Neuroscience (Section Anatomy), University Medical Center Groningen, University of Groningen, Antonius Deusinglaan 1, Box 196, 9700 AD Groningen, The Netherlands

**Keywords:** Sexual behavior, MRI, Connectivity, Wanting, Liking, Inhibition

## Abstract

**Purpose of Review:**

The purpose of this study is to provide a comprehensive summary of the latest developments in the experimental brain study of human sexuality, focusing on brain connectivity during the sexual response.

**Recent Findings:**

Stable patterns of brain activation have been established for different phases of the sexual response, especially with regard to the wanting phase, and changes in these patterns can be linked to sexual response variations, including sexual dysfunctions. From this solid basis, connectivity studies of the human sexual response have begun to add a deeper understanding of the brain network function and structure involved.

**Summary:**

The study of “sexual” brain connectivity is still very young. Yet, by approaching the brain as a connected organ, the essence of brain function is captured much more accurately, increasing the likelihood of finding useful biomarkers and targets for intervention in sexual dysfunction.

## Introduction

Recent years have seen spectacular developments in the field of human brain imaging (neuroimaging) that allow researchers to analyze human brain structure and function in greater detail than was ever possible. These neuroimaging approaches have begun to be applied to the study of human sexual behavior as well. Given the prevalence of idiopathic sexual dysfunctions, this development is positive, but for sex researchers or sexologists not trained to deal with brain data, it can be difficult to get a grip on the wealth of often complex results. In this review, we provide a comprehensive summary of the latest developments in the experimental brain study of human sexuality, with a focus on the sexual response. We will argue that brain connectivity approaches hold the highest promise to provoke breakthroughs regarding the mechanisms that govern functional and dysfunctional human sexual responding.

## From Activity to Connectivity

“Neuroimaging” applies to the use of various techniques to visualize the structure and function of the nervous system. This review almost exclusively deals with results obtained by magnetic resonance imaging (MRI). Structural MRI provides information about the size, shape, and integrity of gray (clusters of cell bodies, e.g., in the cortex) and white (bundles of axons) matter. Analytic methods such as voxel-based morphometry (VBM) can provide reliable estimates of local gray and/or white matter volume differences, either within or between subjects. Diffusion tensor imaging (DTI) is an important structural MRI protocol that can reconstruct a three-dimensional structural map of the white matter tracts (the structural connections) in the brain. Quantitative meta-analyses can combine many data sets to make more reliable inferences about morphological brain features in large populations. An example of this is a study on 1400 human brains from four different datasets that could not substantiate the idea of a clear sexual dimorphism in the human brain [1•].

Functional MRI enables the detection of neural activity over time, typically related to a task, group, physiological or psychological parameter, or individual trait, resulting in functional localization (activation). Again, quantitative meta-analysis methods such as activity likelihood estimation can combine data of multiple activation studies and distill the most robust patterns of activation—those that are likely to resemble functional networks [2, 3••].

Analysis of functional interaction and communication within the brain is termed “functional connectivity” and is essentially calculated as correlations between neural activities of distinct areas. Functional connectivity can be measured for task-based fMRI data, but also for so-called resting state data. The latter does not require intrusive tasks or paradigms that might keep potentially interesting subject groups (e.g., adolescents) from being studied with regard to their sexual brain function. There are different methods that can analyze functional connectivity; some are model-based, such as psychophysiological interaction analysis (PPI) analysis, which can evaluate a more or less specific connection under different task conditions and/or between groups, whereas others like independent component analysis require no task performance and typically can evaluate larger networks or more networks simultaneously [4, 5]. Brain networks that are consistently found in functional connectivity studies, either in the resting state or during task execution, include the default mode network, visual network, sensory/motor network, and task-positive network [6••]. As an example, a study using resting state study found that women had stronger functional connectivity in parts of the default mode network than men did and that the menstrual cycle did not modulate this connectivity. It was concluded that transient activating effects of gonadal hormones could not account for the sexual dimorphism in functional connectivity [7]. Granger causality analysis and dynamic causal models can also provide information about the direction of communication between brain areas [8]. This directed communication between brain areas is called “effective” connectivity.

The most recent analytic developments in neuroimaging aim to capture whole-brain functionality by using tools from the field of network science [9••]. The premise is that the central nervous system behaves as a network, or a system, that tries to achieve an optimal balance between local specialization and global integration. If a network has both properties, it is said to have a small-world organization, and unless there is a severe neurological condition, this usually applies to human brains [10, 11]. However, within a small-world organization, the balance might be shifted towards local specialization or global integration. Graph analysis methods can provide a detailed analysis of this small-world organization, for instance by investigating the number and location of network hubs (areas that function to integrate network activity). At least in theory, graph analysis is capable of providing the most profound insights into neural mechanisms contributing to human sexuality.

## Modeling Sex

**Fig. 1:**
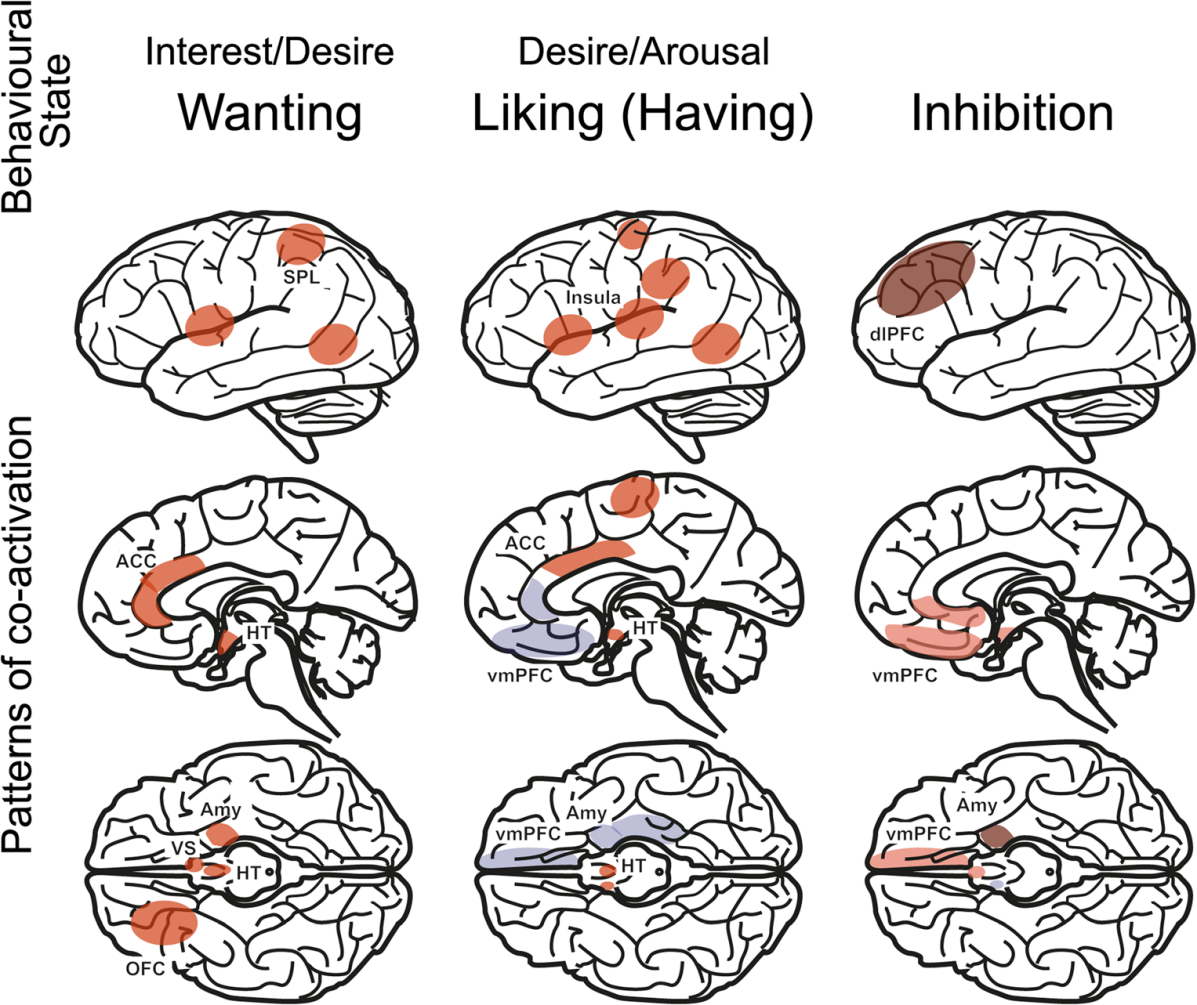
The human sexual pleasure cycle. Brain areas relevant to this review are depicted per phase (red: increased brain activity; blue: decreased brain activity). Inhibition can be physiological (pink shading) or deliberate (brown shading). Abbreviations: ACC, anterior cingulate cortex; Amy, amygdala; dlPFC, dorsolateral prefrontal cortex; HT, hypothalamus, OFC, orbitofrontal cortex; SPL, superior parietal lobule; vmPFC, ventromedial prefrontal cortex; VS, ventral striatum (Figure uses information from [3••, 13])

The term “sexual response” refers to the set of behaviors and functions directly related to sexual stimulation and the pursuit of a sexual goal [12]. Models of the human sexual response aim to provide a template to study and compare a variety of sexual responses, relatively independent of other sexuality characteristics. An example of this is the human sexual pleasure cycle [13, 14•]. This model (Fig. 1)—which underlines the significance of external stimulation next to that of the internal “drive” state (incentive motivation theory) [15, 16]—distinguishes the phases wanting sex, liking sex (or having sex), and inhibiting sex. Sexual orientation, sexual preference, and gender identity are then seen as elements determining what kind of stimuli trigger the sexual pleasure cycle. Clinically, this fits with a distinction between sexual dysfunction (i.e., a problem with the sexual response, e.g., erectile dysfunction) and paraphilia (i.e., an atypical sexual preference, e.g., pedophilia). The use of a model like this facilitates comparison between neuroimaging studies that try to model different elements of the sexual response, while allowing different (neuroscientific) explanations and mechanisms for sexual responsiveness.

## Overview of Recent Neuroimaging Studies on Human Sexuality

**Fig. 2:**
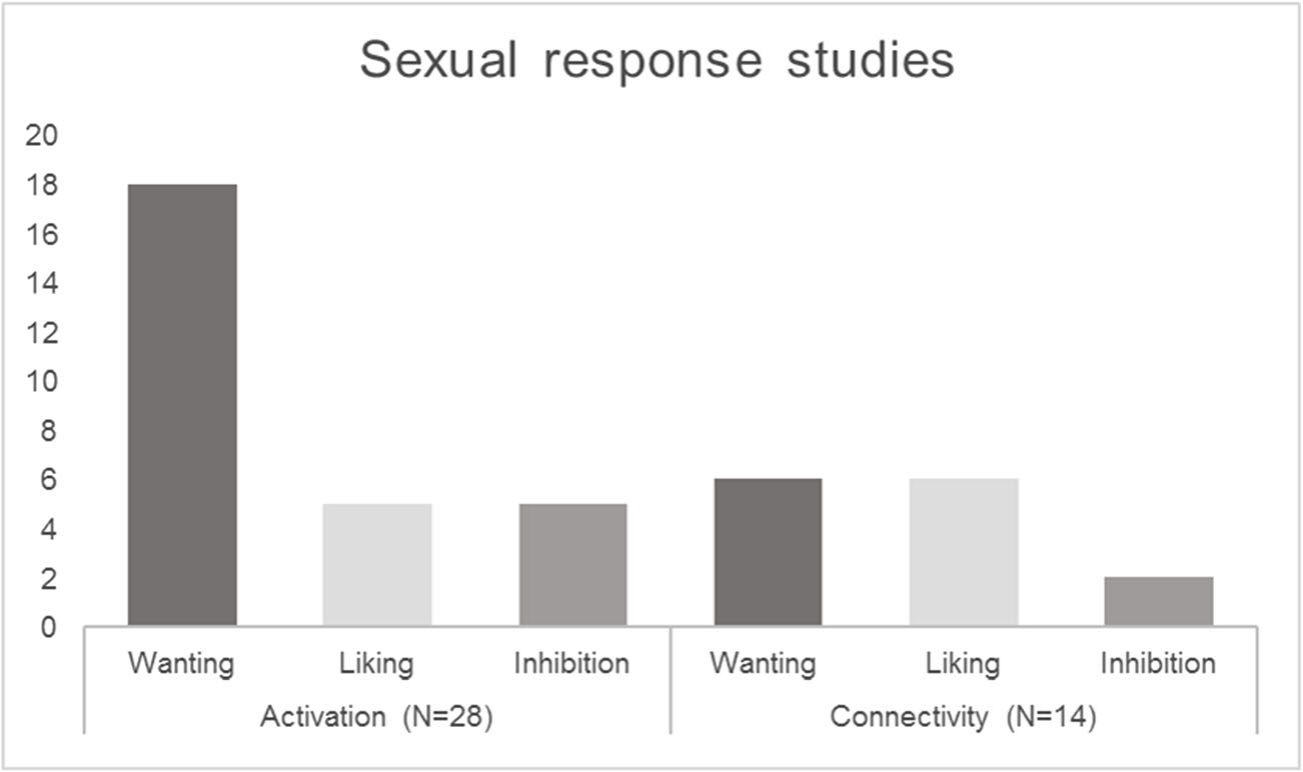
Overview of neuroimaging studies on the sexual response from the period of 2012 to 2017. Studies were categorized by phase of the sexual response cycle investigated (wanting, liking, and inhibition) and by methodology (activation vs. connectivity approaches)

We reviewed relevant human neuroimaging studies that were published in the period 2012–2017, distinguishing studies representing the sexual response itself and factors involved in triggering a response (sexual orientation, preference, or gender identity). Regarding the sexual response category, we distinguished studies representing wanting, liking, and inhibition phases. Studies were further categorized according to their methodology, i.e., whether they employed analytic approaches focusing on separate activated brain areas, or more sophisticated methods analyzing brain connectivity and networks (see previous section). This rough categorization showed that in the domain of the sexual response, about twice as many neuroimaging studies were conducted than in other domains of human sexuality, but also that the relative contribution of connectivity studies was greater in the latter. Furthermore, within the sexual response domain, it is obvious that most of current research efforts are concentrated on the wanting phase, but that connectivity approaches are relatively more common in experiments on the liking phase of the sexual response (Fig. 2).

## Current Status of Human Sexual Response Neuroimaging

Systematic reviews of experimental brain imaging studies of the human sexual response reveal phase-dependent patterns of brain activity (Fig. 1) [3••, 13, 14•, 17]. In their review, Georgiadis and Kringelbach describe a “sexual wanting pattern” including the occipitotemporal cortex, superior parietal lobule, ventral striatum (VS), amygdala/hippocampus, orbitofrontal cortex (OFC), anterior cingulate cortex (ACC), and anterior insula, and a “sexual liking pattern,” including the hypothalamus, anterior and posterior insula, ventral premotor cortex, middle cingulate cortex, and inferior parietal lobule [14•]. Using different terms for basically the same distinction, very similar patterns were identified by Poeppl and colleagues performing a quantitative meta-analysis on psycho- and physiosexual elements of the sexual response [3••]. By and large, a sexual response involves very similar brain activation patterns across sexual preferences and gender groups, as long as preferred sexual stimuli are used [18, 19]. This pattern was refined by a recent meta-analysis, showing a largely consistent pattern across gender groups with statistically significant gender differences mainly in subcortical areas [20]. In addition, there is some indication that phase-dependency in brain response patterns over the course of the sexual response is less marked in women than it is in men [21]. Nevertheless, the stability of the visually evoked sexual wanting pattern was confirmed by scanning subjects on two occasions separated by 1–1.5 years and showing that the brain response was very similar over time [22]. Furthermore, sexual wanting and liking brain response patterns reflect (parts of) known functional brain networks [6••]. Thus, we conclude that these patterns are robust and should be able to provide a solid basis from which sexual response-related brain connectivity can be studied.

More than before, experimental designs are being developed that can avoid confounds caused by participant reaction manipulation. Some studies use subliminal (i.e., below the threshold of consciousness) presentations of sexual stimuli, eliminating elaborate cognitive processing [23]. A novel approach involves adding cognitive loading (mental rotation task) to a visual sexual stimulation design to decrease the likelihood of cognitive reaction manipulation [24]. Such approaches may eliminate unwanted effects of, for instance, adherence to cultural standards on sexual responding.

### Wanting Sex: Non-connectivity Approaches

Neuroscientific interest in the sexual wanting domain is increasingly narrowing down on sexual desire extremes. Several studies using visual sexual stimulation have shown that (perceived) hypersexual behavior (aka compulsive sexual behavior, sexual addiction, or problematic pornography use) is correlated with alterations in neural activation patterns [25–32] and regional brain volume [33•, 34], particularly in areas of the sexual wanting network [14•]. Increased activity to sexual cues has been demonstrated in the VS [25, 27] and also in the amygdala in hypersexual men [25, 27, 28], which is suggestive of sexual cue sensitization. This is sometimes taken to support the addiction theory of hypersexuality [35]. Other studies, however, showed negative correlations between sexual cue-induced brain activity and hypersexual symptom severity, suggesting the involvement of different phenomena that are seemingly incompatible with addiction, like response extinction or emotional downregulation [26, 28–30, 34]. These data may not be mutually exclusive. For instance, men with hypersexuality may be both sensitized to sexual cues or contingencies (a feature of addiction) and more easily lose interest or self-regulate if there is no possibility to advance the sexual response (as a learned adaptation). Indeed, in a paradigm with repeated exposure of cues predicting the presentation of a pornographic picture or a monetary reward, cue-induced activity in the ACC decreased faster with repeated exposure in men with hypersexuality—but only for the sexual cues [26].

At the other end of the spectrum, sexual interest/arousal disorder is associated with structural and functional alterations in the sexual wanting network, especially in areas like the ACC, VS, and amygdala, suggesting decreased sexual cue sensitivity [36]. Rupp and colleagues showed that in postpartum women, amygdala responses to emotional pictures (including erotic pictures) was suppressed, indicating decreased sensitivity to emotional salience during the postpartum period [37]. A resting state fMRI study suggested that antidepressant use is associated with altered functional connectivity within the sexual wanting network, especially with regard to the connectivity of the (extended) amygdala. In this study, amygdala connectivity profile prior to antidepressant use reliably predicted if a subject was going to be vulnerable or resilient to antidepressant-related sexual dysfunction [38].

The “sexual wanting network” can be recruited by a range of salient non-erotic stimuli as well [14•], including negative ones [39]. The question then becomes how generic and specific functions work together within this network to produce a distinct *sexual* interest. Although this question is far from being answered, interesting new insights have been published, mostly on the VS. For instance, VS responses to food and erotic images predicted individual differences in bodyweight and sexual activity, respectively, 6 months later [40]. Another study reported that differences in VS activation for monetary versus erotic cues could be explained by their relative motivational value [41•]. Hence, the VS might signal values for different reward types, but the neural responses for each reward type are unique and are influenced by their salience for a given person. Indeed, relative to healthy controls, men with hypersexuality show stronger VS activity for preferred relative to non-preferred visual erotica [32]. Another area of interest in this context is the OFC, because reward subtypes are processed in different OFC subregions [42]. While primary rewards (like erotic stimuli) activate the OFC posteriorly, secondary rewards (like money) activate a more anterior portion [43]. The OFC is thus a prime candidate to further the study how the brain produces distinct sexual interest and feelings.

Sexual responsiveness shows normal short-term and long-term variability. This has been studied mostly in the context of the sex steroid milieu. Contrary to the biological adage that fertility status drives sexual responsivity, no consistent pattern emerges from studies trying to find a relationship between visual stimulation-induced brain activity and menstrual cycle phase [21]. However, Abler and colleagues included an expectancy element in their study and found that, in regularly cycling women, the predicting stimulus (conditioned cue) activated the ACC, OFC, and parahippocampal gyrus more strongly during the luteal phase than the follicular phase. Activation in these areas was stronger in regularly cycling women, as compared to those on oral contraceptives [44].

Testosterone is seen as the gonadal hormone most pertinent to human sexual responsiveness [45, 46]. Indeed, brains of genetic men without androgen function (complete androgen insensitivity syndrome, “46XY women”) responded in a typical female-like fashion to visual erotic stimulation, that is, similar to male controls but at weaker strength [47]. Because in both 46XY and genetic women, there is less central testosterone function than in men; it was concluded that testosterone rather than genetic sex determines brain activity patterns during sexual stimulation. Yet, a DTI experiment studying brain structure in transgender and cisgender women and men found white matter variation that could not be accounted for by differences in testosterone function. Trans people exhibited white matter values midway between male and female cisgender controls, despite gonadal hormone levels being either typically male or female (depending on whether they were transgender women or transgender men) [48].

### Wanting Sex: Connectivity Approaches

Functional connectivity within the sexual wanting network has recently been investigated using the PPI approach, mainly in the context of (perceived) hypersexuality. Men with hypersexuality and controls both show increased functional connectivity of the ACC with both the right VS and right amygdala when viewing erotica, but the strongest positive correlation with reported sexual desire was found for ACC-subcortical connectivity in hypersexuality [25]. After many repetitions of sexual stimulation, functional connectivity of the ACC with the right VS and with the bilateral hippocampus was stronger in men with hypersexuality than in controls. Intriguingly, this increased functional *connectivity* within the sexual wanting network occurred in the presence of decreased ACC *activity* [26]. This could signify a habituation effect, but more research is required to explore this phenomenon. Another study used a design with cues predicting pornographic or non-erotic stimuli and found decreased functional connectivity between the VS and ventromedial PFC for men with hypersexuality compared to controls [28]. Since altered VS-prefrontal coupling has been associated with impulsivity control, substance abuse, and pathological gambling [49–51], these findings could be an indication of inhibition impairment in men with hypersexuality. Two other studies employed a resting state design, showing that (*i*) reported hours of watching pornography (per week) are negatively correlated with resting state connectivity between the right caudate nucleus and left dorsolateral PFC and (*ii*) subjects diagnosed with compulsive sexual behavior have decreased functional connectivity between the left amygdala and bilateral dorsolateral PFC [33•, 34]. These studies indicate that increases in sexual behavior are marked by altered prefrontal control mechanisms. Together, these connectivity studies strengthen the assumption that the “sexual wanting” pattern identified by activation studies is indeed the resemblance of a true functional network, because a subset of its constituent brain areas alters their communication when sexual incentives are presented, while the strength of this interaction reflects the sexual behavioral phenotype. Fronto-striatal connectivity and VS connectivity hold high promise as research avenues into the fundamentals of (aberrant) sexual wanting.

### Liking Sex

Brain imaging paradigms employing stronger and more prolonged visual sexual stimulation (for example, porn movies), or tactile genital stimulation, are likely to model (elements of) having sex (e.g., evoke physiological genital responses and sexual liking). As indicated earlier, this phase recruits a brain network that is relatively distinct from that recruited during wanting sex, and this is especially so in men [3••, 13, 14•, 20]. Liking sex has also seen more studies focusing on brain connectivity than wanting sex has (Fig. 1).

One disorder that is currently receiving particular attention is psychogenic erectile dysfunction (pED). This condition has been associated with increased or decreased gray matter volume in many brain areas, including those belonging to sexual wanting and liking networks [52, 53•]. It has also been associated with persistent sexual wanting network activation (superior parietal lobule specifically), possibly resulting in a failure to shift to the next phase of the sexual response cycle [54]. Interestingly, pED is now predominantly being studied with structural or resting state neuroimaging research paradigms, contrary to other sexual disorders that are dominated by task-based paradigms. Altered functional connectivity within and beyond sexual wanting and liking networks has been identified. For instance, the right lateral OFC was found to have aberrant structural connectivity with areas in the parietal lobe in pED [53•]. In a resting state fMRI study, pED subjects showed altered functional connectivity of the right anterior insula (an area integral to interoception and emotion regulation) with the dorsolateral PFC and right parietotemporal junction, compared to controls [55]. This indicates that pED may come with an abnormal representation of bodily states (including erection) and/or excessive inhibition control. Interestingly, when subjects viewed a porn movie for the duration of the experiment (instead of resting), reduced functional connectivity of the right insula was also found in individuals with pED relative to healthy volunteers [56]. Even though the experimental paradigms differ, the results seem congruent, again involving components of both wanting and liking networks that also show structural degradation in pED [53•].

None of the studies discussed so far have considered whole-brain connectivity. In fact, the first study to do this was published only 2 years ago. Zhao and colleagues applied graph analysis methods to structural data to study diverging brain connectivity profiles in pED subjects [57••]. As expected, the whole-brain connectivity profile of pED subjects and healthy subjects had a small-world organization characterized by both networks for local specialization and global integration. However, in pED, the balance was shifted towards local specialization, possibly resulting in poorer integration of network activity. Indeed, fewer hubs (integrating areas) were identified in pED than in controls, indicating overall poorer global integration.

Genital stimulation is the primary source of sexual pleasure (liking) in the brain and is a key contributor to sexual arousal [13]. Yet, very little is known about the brain’s role in sexual development of genital sensations. Some new insights are provided by research in spina bifida patients who underwent a surgical reinnervation of their lifelong insensate penis to improve their sexual function. Stimulation of the glans penis (reinnervated by a groin nerve) and the intact groin area (contralateral to the area that provided the donor nerve) activated the same area of the primary somatosensory cortex, as expected. However, primary somatosensory cortex was functionally connected with the MCC and operculum-insular cortex during penis stimulation, but not during groin stimulation [58]. Wise et al. studied to what extent brain activation overlaps or differs for both physical and imagined genital stimulation in women [59]. One of the more interesting results is that imagined dildo stimulation activated hippocampus/amygdala, insula, VS, ventromedial PFC, and somatosensory cortices more than imagined speculum stimulation. Another recent study in masochists showed decreased functional connectivity of the parietal operculum with the bilateral insulae and operculum when they received painful stimuli in masochistic context, indicating a network for pain modulation in favor of sexual arousal [60]. Even when candidate areas have been suggested, clearly more work is needed to identify the key areas that govern not only the sexual interpretation of genital sensation in relation to context, but also the transition of genital to sexual sensations in normal sexual development.

### Inhibiting Sex

From a behavioral point of view, the potential to inhibit or control a sexual response is equally critical as being able to respond sexually. Thus, in the brain, there must be a continuous interplay between systems that promote approach and systems that promote avoidance. A more or less consistent finding is that prefrontal areas tend to show exaggerated activity in subjects with hyposexual behavior [61–63]. However, breast cancer survivors who report distress about their loss of sexual desire showed *reduced* activity in the dorsolateral PFC and ACC when viewing pornographic pictures, compared to non-distressed breast cancer survivors [64]. This result seems counterintuitive, but chronic stressors are associated with prefrontal hyporegulation of subcortical areas [65]. Clinical findings confirm that prefrontal function needs to be within an optimal range for sex to function normally [66], illustrating the very important point that normal brain function requires optimal balancing of brain systems.

Victor and colleagues performed an interesting fMRI study focusing on the VS-amygdala balance as an index of the individual trait to inhibit sexual responding [67••]. Their hypothesis was that VS responding to appropriate sexual stimuli is only half of the story; in order for a sexual response to advance, the amygdala should also deactivate to “release the brake.” This is in line with studies showing decreased medial temporal lobe activity during high sexual arousal (e.g., see [14•]). Interestingly, high VS and low amygdala activity during a non-erotic impulsivity test was indeed found to predict a higher number of sex partners 6 months after the study, but in male participants only; in women, the highest number of new sex partners was predicted by a combination of high VS and amygdala activity [67••]. Importantly, VS and amygdala activity might also reflect a specific negative appreciation of sexual stimulation. In a recent fMRI study which included an implicit association test, women viewed images of explicit penetrative sex. Contrary to what might be expected, VS activity (and the basal forebrain-amygdala continuum) did not reflect approach or positive interest; instead, those subjects that showed the strongest automatic avoidance of extreme porn had the strongest porn-induced VS response [68•]. Together, these findings clearly demonstrate that detecting a salient sexual stimulus is not sufficient to advance a sexual response, but rather, that sexual response results from a complex interplay between approach and avoidance, the neural mechanisms of which are only beginning to be unveiled.

## Conclusion and Future Directions

Human sexuality does not rely on a single “sex nucleus.” Rather, it involves many—sometimes quite generic—brain functions including those for arousal, reward, memory, cognition, self-referential thinking, and social behavior. As clearly shown in this review and elsewhere [3••, 14•, 17], the brain areas that have been associated with human sexuality are spatially remote. From this point of view, studying the connectivity of the brain is much more intuitive than studying separate “activations,” and in fact, studying the nature of the connectivity between brain regions has been a common practice in animal models of human sexual behavior for many decades already (see e.g., [46]). Every fraction of a second, billions of neurons “talk” to each other by virtue of an unthinkable wiring creating even more complex neural networks. It is by understanding how these networks operate—alone, but preferably in conjunction with each other—that we can begin to understand the neural mechanisms that critically regulate human sexual function and that can account for non-organic sexual dysfunction. Currently, the urgency to take such an approach seems more pertinent in other fields of sexuality research, like gender identity/transsexuality and child sexual offending. For instance, a recent study used structural MRI data to define regions with gray matter deficits in pedophilia and then assessed a reliable functional connectivity profile of these areas using a large brain database (data from 7500 brain experiments were used). It turned out that morphologically altered areas in pedophilia are functionally connected primarily with areas important for sexual responsiveness, i.e., areas of the sexual wanting and liking networks [69••]. This is strongly suggestive of a situation where a functional sexual response is connected to—or controlled by—brain regions with significant morphological deficits. As another example of more sophisticated application of neuroimaging to the study of human sexuality, a recent study used graph analysis to show that, relative to cisgenders, transgender people have a stronger local specialization of their somatosensory network, characterized by more and stronger local connections [70]. Most likely, this underlies their differential body perception. By approaching the brain as a connected organ, studies such as these capture the essence of brain function much more accurately, increasing the likelihood of finding useful biomarkers and targets for intervention. We strongly encourage that such methods be used more to study the human sexual response, because accepting that conditions like sexual pain/penetration disorder, sexual interest /arousal disorder, hypersexual complaints, premature ejaculation, persistent genital arousal disorder, and anorgasmia originate in the brain is not enough; sexual dysfunctions are complex, multidimensional, and multifactorial and by their very nature, suitable to be studied from a “connectivity” perspective.
